# The cAMP-PKA pathway-mediated fat mobilization is required for cold tolerance in *C*. *elegans*

**DOI:** 10.1038/s41598-017-00630-w

**Published:** 2017-04-04

**Authors:** Fang Liu, Yi Xiao, Xing-Lai Ji, Ke-Qin Zhang, Cheng-Gang Zou

**Affiliations:** State Key Laboratory for Conservation and Utilization of Bio-Resources in Yunnan, Yunnan University, Kunming, Yunnan 650091 China

## Abstract

Low temperature has a great impact on animal life. Homoiotherms such as mammals increase their energy expenditure to produce heat by activating the cAMP-protein kinase A (PKA)-hormone-sensitive lipase (HSL) pathway under cold stress. Although poikilothermic animals do not have the ability to regulate body temperature, whether this pathway is required for cold tolerance remains unknown. We have now achieved this using the genetically tractable model animal *Caenorhabditis elegans*. We demonstrate that cold stress activates PKA signaling, which in turn up-regulates the expression of a hormone-sensitive lipase *hosl-1*. The lipase induces fat mobilization, leading to glycerol accumulation, thereby protecting worms against cold stress. Our findings provide an example of an evolutionarily conserved mechanism for cold tolerance that has persisted in both poikilothermic and homoeothermic animals.

## Introduction

Low environmental temperature can have a significant impact on physiological processes in a variety of living organisms. Non-freeze cold exposure (i.e. chill) may induce cytotoxicity, such as collapse of ion homeostasis^1^, lipid structure changes to a more rigid gel phase^2^, and increased oxidative stress^3^. Cold insults accumulate over time, resulting ultimately to organism death. Thus, many organisms have developed adaptive mechanisms to survive in cold conditions.

In mammals, cold induces thermogenesis, which mainly consists of shivering and non-shivering thermogenesis^4, 5^. Shivering is uncomfortable and cannot be sustained over prolonged periods. Non-shivering thermogenesis mainly occurs in brown adipose tissue, where β3-adrenergic receptors coupled to G proteins of the Gs subtype induces cAMP formation. cAMP in turn activates protein kinase A (PKA), thereby stimulating fat hydrolysis via activation of hormone-sensitive lipase. Fat hydrolysis ultimately leads to the liberation of glycerol and free fatty acids. The latter is involved in the physiological activation of uncoupling protein 1 (UCP1), allowing mitochondria to convert ATP to heat, rather than to energy.

In poikilotherms, endothermy has been observed in several species of fish, including the billfish, swordfish, and the butterfly mackerel^6^. These endothermic fishes possess thermogenic organs, which have evolved from skeletal muscle and warm the brain and eyes up to 20 °C above ambient water temperature^7^. In contrast, poikilothermic invertebrates cannot raise and sustain their body temperature significantly above the ambient temperature by endogenous heat production. However, these poikilotherms can make physiological and behavioral changes to cope with cold stress. For instance, in many organisms, cold conditioning frequently results in the modification of membrane lipids, often by increasing the proportion of unsaturated fatty acids in the phospholipids^8, 9^. As low temperatures lead to lipid structure changes to a more rigid gel that impairs vital membrane functions, increased lipid unsaturation may decrease the temperature at which this transition occurs^10^. In response to cold stress, a variety of metabolites, including glycerol^11, 12^, sugars (e.g. trehalose, fructose, glucose, and sucrose)^13^, and free amino acids (e.g. proline and alanine)^13, 14^ are elevated in many poikilothermic invertebrates. These metabolites are thought to be involved in enhanced resistance to cold stress.

The cAMP-PKA signaling pathway plays a major role in physiology, such as the control of metabolism, apoptosis, and cell differentiation^15^. The activation of the cAMP-PKA pathway has been observed in poikilothermic organisms at low temperatures^16–18^. For instance, freezing can lead to a rapid increase in cAMP levels and an increase in the percentage of the free catalytic subunit of PKA in the liver of wood frogs^16, 17^. PKA in turn promotes the conversion of glycogen to glucose, which is believed to function as a cryoprotectant^17^. In yeast, the cAMP-PKA pathway regulates a large number of gene expressions under low temperature conditions^18^. These results indicate that this signaling pathway probably plays an important role in cold adaptation. In this study, using a genetically tractable metazoan *Caenorhabditis elegans*, we found that genetic inactivation of the core components in the cAMP-PKA pathway enhanced the susceptibility to cold stress. PKA up-regulated the expression of a hormone-sensitive lipase *hosl-1*. The lipase promoted fat mobilization, resulting in an increase in glycerol levels, which is required for cold resistance.

## Results

### The cAMP-PKA pathway positively regulates cold stress

**Figure 1:**
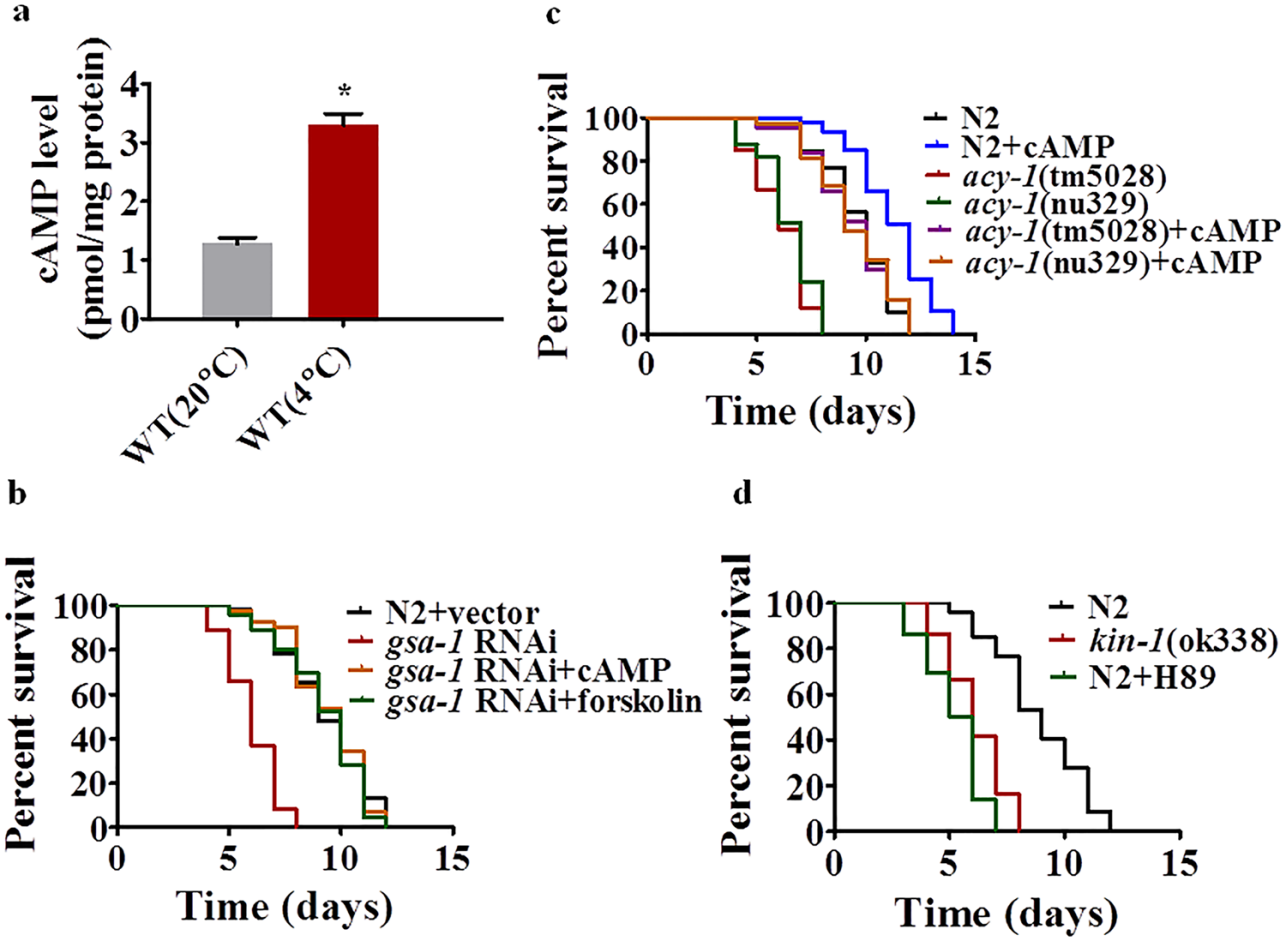
The cAMP-PKA signaling cascade is involved in cold tolerance. (**a**) Exposure to cold temperature (4 °C) led to an increase in cAMP levels in wild type worms (WT). These results are means ± SD of three experiments. *P < 0.05. (**b**) *gsa-1*(*RNAi*) reduced the survival rate of worms at 4 °C. Addition of cAMP (5 mM) or forskolin (0.5 mM) to *gsa-1*(*RNAi*) animals recovered the resistance to cold stress. *P* < 0.001 relative to WT with empty vector. (**c**) *acy-1*(*tm5028*) or *acy-1*(*mu329*) mutant worms exhibited enhanced susceptibility to cold stress. Addition of cAMP (5 mM) to *acy-1* mutants recovered the resistance to cold stress. *P* < 0.01 relative to WT. (**d**) *kin-1*(*ok338*) mutant worms exhibited hypersensitivity to cold stress. H89 (10 μM) reduced the survival rate of WT worms upon cold exposure. *P* < 0.001 relative to WT.

In *C*. *elegans*, the Gα protein GSA-1, which is orthologous to the mammalian Gsα, regulates nematode locomotion through ACY-1 to produce cAMP^19^. The *C*. *elegans* PKA catalytic and regulatory subunits are encoded by *kin-1* and *kin-2*, respectively. The binding of cAMP to KIN-2 leads to its dissociation from the inactive holoenzyme and the release of active KIN-1. To address the role of the cAMP-PKA pathway in cold tolerance, we first determined the cAMP levels in young adult worms after transferring them directly from an optimal growth temperature (20 °C) to a low temperature (4 °C). Cold stress resulted in a significant increase in the cAMP levels at 12 h after exposure to 4 °C (Fig. 1a). To test the role of *gsa-1* in survival of worms during cold stress, *gsa-1* was silenced by RNAi. *gsa-1*(*RNAi*) reduced survival of worms during cold stress (Fig. 1b). In contrast, the survival rates of *gsa-1*(*ce94*) or *gsa-1*(*ce81*) gain of function mutants were higher than those of wild type (WT) worms (Supplementary Fig. 1). Meanwhile, exogenous application of either cAMP (5 mM) or forskolin (0.5 mM), an agonist of adenylatecyclase^20^, to *gsa-1*(*RNAi*) worms significantly restored the resistance to cold stress (Fig. 1b).

Of all four adenylyl cyclases in *C*. *elegans*, only mutations in *acy-1*(*tm5028*) or *acy-1*(*nu329*) reduced the survival of worms under cold stress (Fig. 1c, Supplementary Fig. 2a). Similar results were obtained in WT worms subjected to *acy-1* RNAi (Supplementary Fig. 2b). Moreover, the addition of cAMP (5 mM) rescued the susceptibility to cold stress in *acy-1*(*tm5028*) or *acy-1*(*nu329*) mutant worms and promoted the survival of WT worms (Fig. 1c).

Finally, we found that *kin-1*(*ok338*) mutant worms were more sensitive to cold stress than WT worms (Fig. 1d). These results were confirmed by *kin-1*(RNAi) (Supplementary Fig. 3). Furthermore, H89 (10 μM), a specific inhibitor of PKA^20^, reduced survival of WT worms under cold stress (Fig. 1d). Conversely, *kin-2*(*ce179*) mutants were more resistant to cold stress than wild-type worms (Supplementary Fig. 4). Taken together, the GSA-1/ACY-1/KIN-1 pathway is required for resistance to cold stress in worms.

### KIN-1 elicits lipid hydrolysis

**Figure 2:**
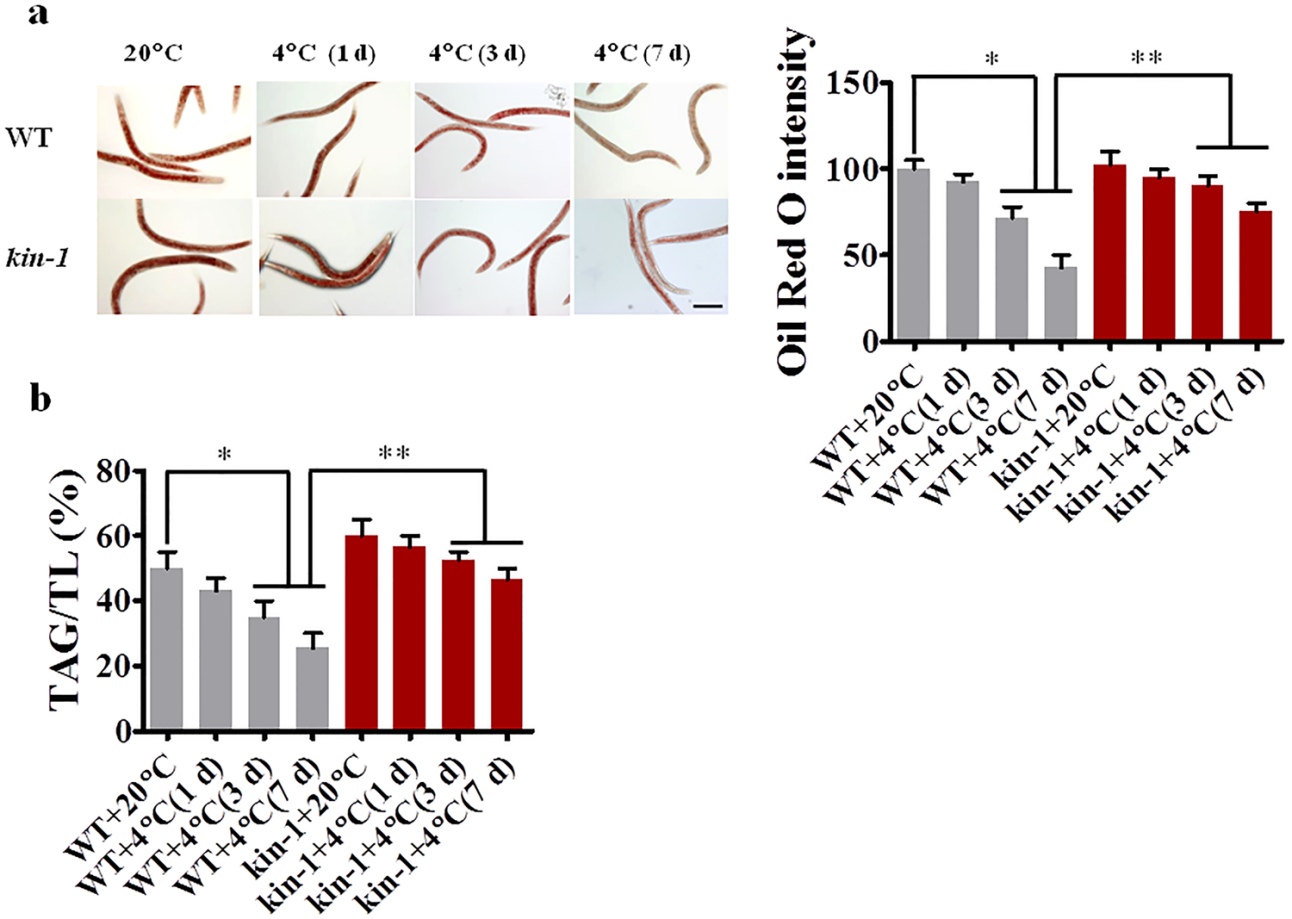
Cold exposure promotes lipid hydrolysis. (**a**) A representative image of lipid droplets using Oil Red O staining. The quantity of lipid droplets in wild type worms (WT) was significantly reduced after cold exposure at 1, 3, and 7 days. However, the mutation in *kin-1*(*ok338*) inhibited cold-induced lipid hydrolysis. *P < 0.05; **P < 0.01. Scale bar, 150 μm. (**b**) The relative triacylglycerol contents were determined by GC-MS. *P < 0.05; **P < 0.01.

In mammals, fat mobilization is required for thermogenesis during cold exposure^21^. To test whether fat mobilization occurs in *C*. *elegans*, we determined the amount of lipid droplets in the intestine by Oil Red O staining. We found that the lipid content was reduced under cold conditions (Fig. 2a). To further confirm these results, we also quantified triglyceride (TAG) content by TLC-GC-MS. We found that the percentage of TAG in total lipids was significantly reduced in WT, but not in *kin-1*(*ok338*) worms, under cold conditions (Fig. 2b). The mutation in *kin-1*(*ok338*) significantly suppressed cold-induced lipid hydrolysis. Interestingly, worms at 4 °C displayed an increase in monounsaturated fatty acid content (Supplemental Table 1), supporting the view that cold exposure leads to an increased proportion of monounsaturated fatty acids^22^. It should be noted that exposure to chill (4 °C) resulted the defects in egg production (Supplementary Fig. 5). Although reproduction probably alters lipid levels in worms, it is unlikely that the alteration in lipid levels during cold stress is due to reproduction. Taken together, these results demonstrate that cold stress promotes lipid mobilization in a KIN-1-dependent manner in *C*. *elegans*.

### HOSL-1 is required for KIN-1-mediated fat hydrolysis

**Figure 3:**
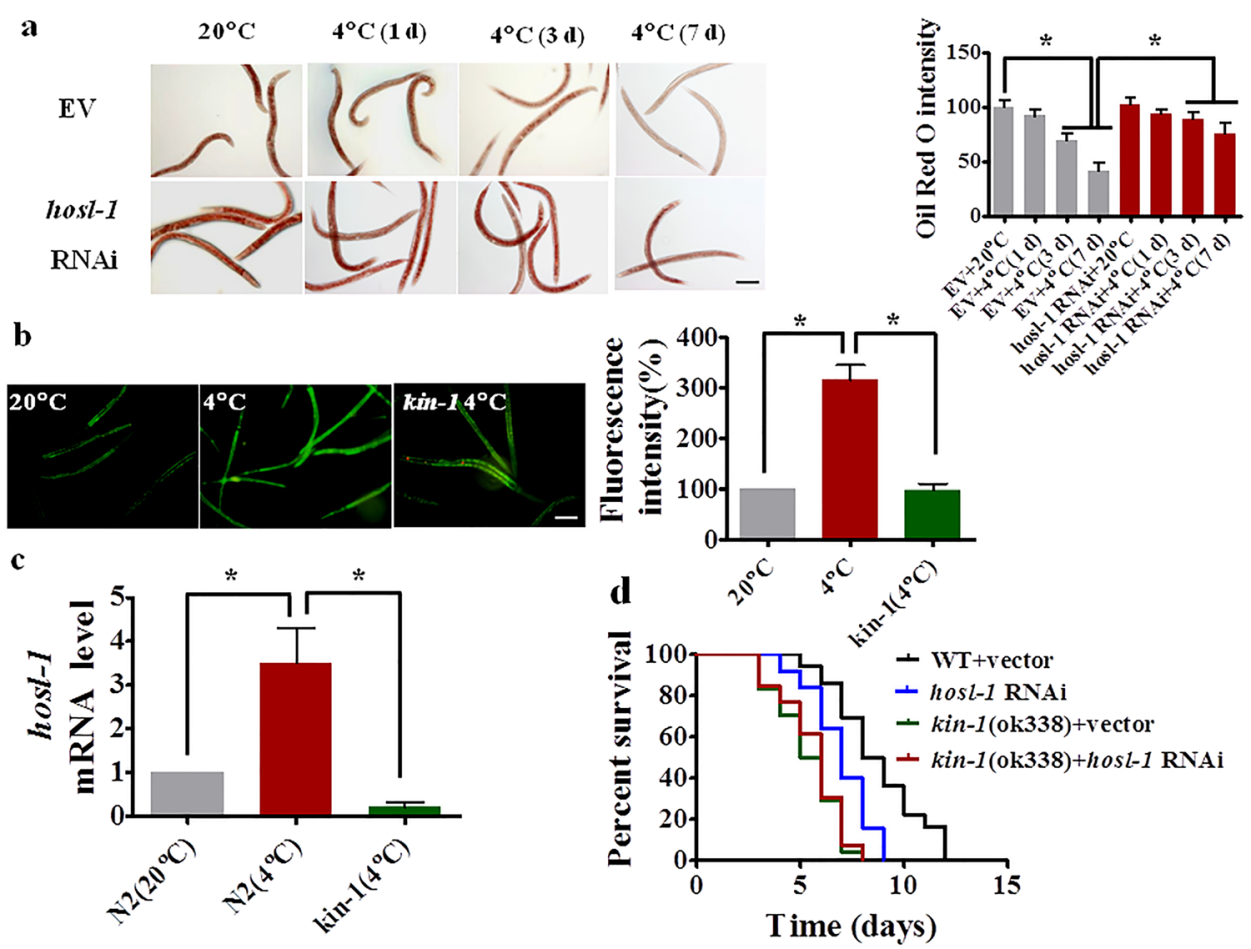
Fat mobilization is mediated by HOSL-1 during cold stress. (**a**) A representative image of lipid droplets using Oil Red O staining. Lipid hydrolysis was significantly inhibited in worms subjected to *hosl-1*(*RNAi*). *P < 0.05. Scale bar, 150 μm. (**b** and **c**) The expression of *Phosl-1*::*gfp* (**b**) or the mRNA levels of *hosl-1* (**c**) were up-regulated at 12 h after cold exposure. The mutation in *kin-1*(*ok338*) inhibited the expression of *hosl-1*. The right panel represents relative GFP fluorescence intensity (**b**). *P < 0.05. Scale bar, 100 μm. (**d**) *hosl-1*(*RNAi*) reduced the survival rate of worms at 4 °C. However, *hosl-1* RNAi did not enhance cold susceptibility of *kin-1*(*ok338*) worms. *P* < 0.001 relative to WT with empty vector.

To identify which gene(s) is involved in fat mobilization, we screened 20 genes in lipid metabolism pathways, which are covered by the Ahringer RNAi library. These genes include 12 class II lipase genes (*lips-1*, *-2*, *-3*, *-4*, *-7*, *-8*, *-9*, *-12*, *-13*, *-14*, *-17*, and *fil-1*), six lipase-related genes (*lipl-1*, *-2*, *-3*, *-4*, *-5*, *-7*), one hormone-sensitive lipase (HSL) homolog gene (*hosl-1*), one Desnutrin/adipose triglyceride lipase gene (*atgl-1*)^23^. We found that *hosl-1*(*RNAi*), but not other genes, significantly inhibited fat hydrolysis under cold conditions (Fig. 3a, Supplementary Fig. 6a and 7–9). These results suggest that HOSL-1 is involved in fat hydrolysis during cold stress.

Using transgenic worms expressing *Phosl-1*::*gfp* ^24^, we found that the expression of *Phosl-1*::*gfp* was up-regulated in worms after 12 h of cold exposure (Fig. 3b). However, the mutation in *kin-1*(*ok338*) abolished the increase in *Phosl-1*::*gfp*. Likewise, using qRT-PCR, we observed that under cold conditions, the mRNA levels of *hosl-1* were significantly elevated in WT, but not in *kin-1*(*ok338*) worms (Fig. 3c). Furthermore, *hosl-1*(*RNAi*) led to enhanced sensitivity to cold stress in WT worms, but not in *kin-1*(*ok338*) background (Fig. 3d). Furthermore, worm overexpression *hosl-1* exhibited enhanced resistance to cold stress (Supplementary Fig. 6b). Taken together, these results are consistent with KIN-1 acting upstream of *hosl-1* to promote fat mobilization and in response to cold stress.

In mammals, PKA exhibits its biological functions through phosphorylating a variety of substrates, such as the transcription factor cAMP-regulatory element-binding protein (CREB)^25^. We thus tested whether KIN-1 regulates *hosl-1* via CRH-1, the *C*. *elegans* CREB orthologue. However, we found that *crh-1*(*RNAi*) did not influence the expression of *Phosl-1*::*gfp* at 4 °C (Supplementary Fig. 10a). Meanwhile, a mutation in *crh-1*(*tz2*) did not alter the mRNA levels of *hosl-1* at 4 °C (Supplementary Fig. 10b). These results excluded a role of CRH-1 in transcriptional regulation of *hosl-1* expression mediated by KIN-1.

### KIN-1/HOSL-1 mediates the production of glycerol, which is required for resistance to cold stress

**Figure 4:**
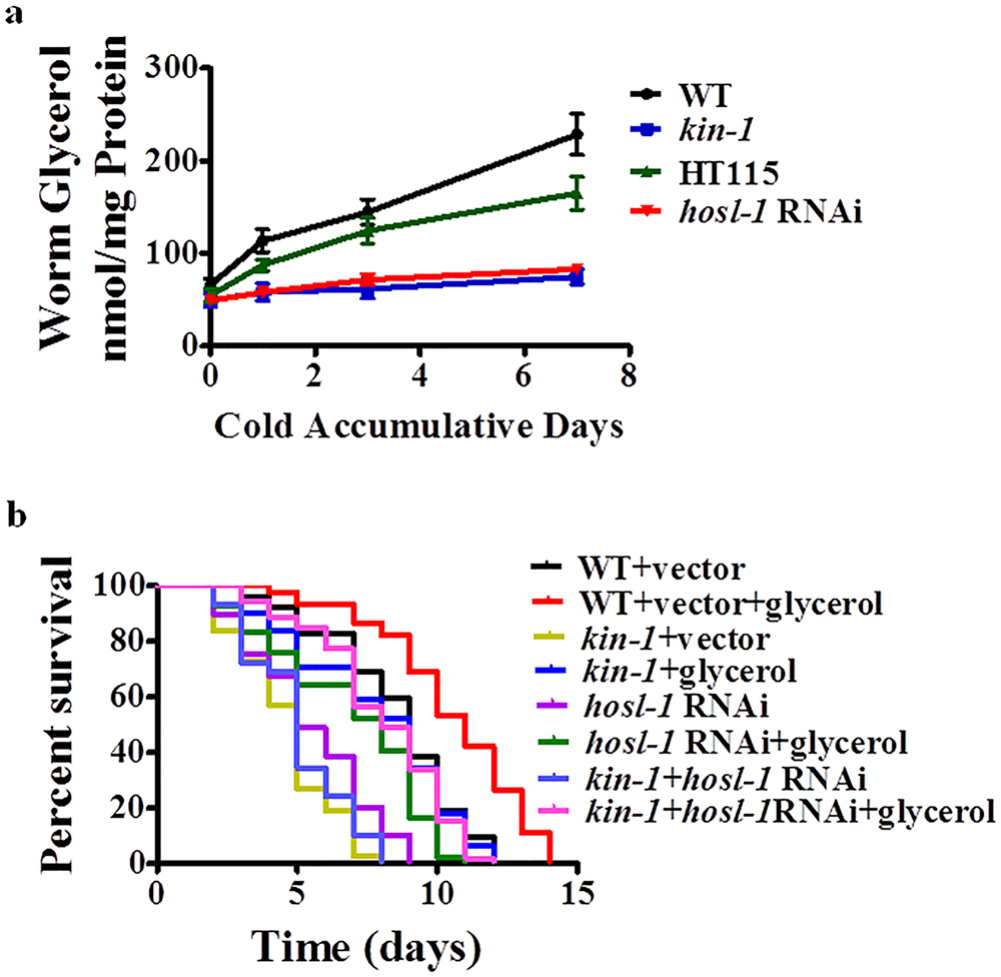
Glycerol accumulation is mediated by KIN-1 and HOSL-1. (**a**) The glycerol contents were elevated after 1, 3, and 7 days of cold exposure in wild type (WT) worms. The mutation in *kin-1*(*ok338*) or *hosl-1*(*RNAi*) inhibited the glycerol accumulation. These results are means ± SD of four experiments. (**b**) Addition of glycerol (5 mM) not only recovered the resistance to cold stress in *kin-1*(*ok338*) or *hosl-1*(*RNAi*) worms, but also enhanced the resistance to cold stress in wild type worms. *P* < 0.01 relative to WT.

To further confirm that cold stress promotes lipid mobilization, we determined the levels of glycerol, which is a product of triglyceride hydrolysis. Indeed, cold stress led to a significant increase in the levels of glycerol in WT worms (Fig. 4a). However, the mutation in *kin-1*(*ok338*) or *hosl-1*(*RNAi*) markedly suppressed the accumulation of glycerol in worms exposed to cold. *De novo* synthesis is one of the main sources of glycerol. We thus test the expression of glycerol-3-phosphate dehydrogenase *gpdh-1*, which catalyzes the rate-limiting step of glycerol biosynthesis, using the transgenic worms expressing *Pgpdh-1*::*gfp*. We found that cold exposure did not influence *Pgpdh-1*::*gfp* (Supplementary Fig. 11). This observation is consistent with the results reported by Lamitina *et al*.^26^ It should be noted that the levels of glycerol were lower in worms fed RNAi bacterium *E*. *coli* HT115 than those in worms fed *E*. *coli* OP50. It is probably due to lower TAG levels in worms fed *E*. *coli* HT115^27, 28^.

Why is fat mobilization beneficial to cold resistance? In homoiotherms, lipid hydrolysis leads to the release of free fatty acids, which in turn activate UCP1 for thermogenesis^4, 5^. However, such a mechanism does not exist in poikilothermic invertebrates. Lipid hydrolysis also produces glycerol. Thus, we speculated that the glycerol is likely involved in enhancing cold resistance. To test this hypothesis, we tested the effect of exogenous glycerol on the survival of worms upon cold exposure. We found that exogenous application of glycerol (5 mM) was not only sufficient to restore the resistance to cold stress in *kin-1*(*ok338*) mutant or *hosl-1* RNAi worms, but also extended the survival of cold stress of WT worms under cold conditions (Fig. 4b).

### PKA in neurons and intestine regulates cold stress

**Figure 5:**
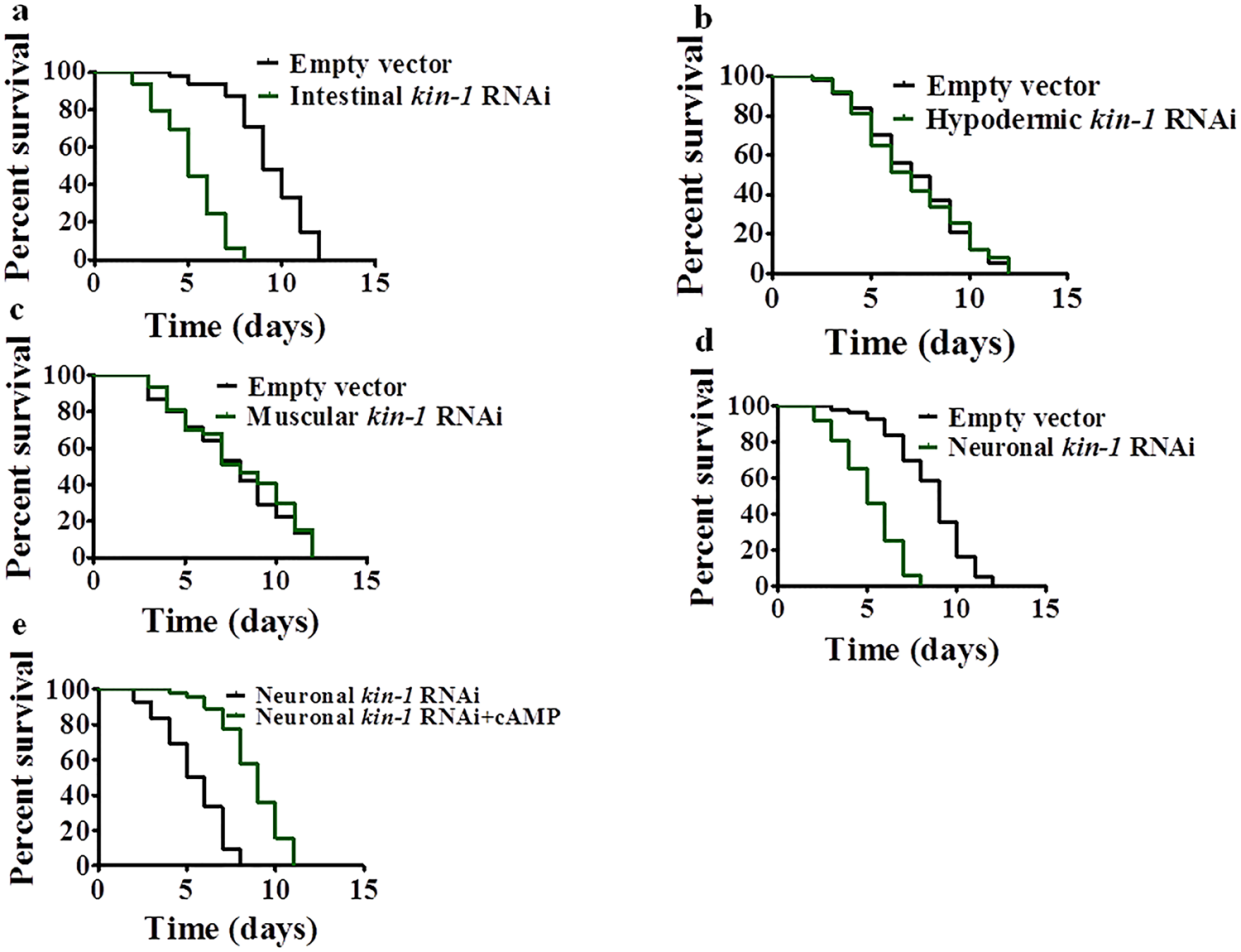
KIN-1/PKA functions in the intestine and neurons during cold stress. (**a**) Intestinal-specific *kin-1* RNAi significantly reduced survival rate of worms at 4 °C. *P* < 0.01 relative to control with empty vector. (**b**,**c**) Hypodermic-(**b**) or muscular- (**c**) specific RNAi had no effect on sensitivity to cold stress. (**d**) Neuronal-specific *kin-1* RNAi significantly reduced survival rate of worms at 4 °C. *P* < 0.01 relative to empty vector. (**e**) cAMP rescued the specific neuronal RNAi *kin-1* phenotype to cold stress. *P* < 0.01 relative to specific neuronal RNAi *kin-1*.

*kin-1* is expressed in the intestine, excretory cell, and all neurons of nematodes^29, 30^. To determine tissue-specific activities of KIN-1 in resistance to cold tolerance, we used the tissue-specific RNAi strains to silence *kin-1* by RNAi in the intestine^31^, hypodermis^32^, and muscle^32^, respectively. We found that intestinal-specific *kin-1*(*RNAi*) reduced the survival of worms during cold stress (Fig. 5a). In contrast, hypodermic-, or muscular-specific *kin-1*(*RNAi*) did not impact on their response to cold stress (Fig. 5b,c). For RNAi silencing in neurons, we utilized TU3401 worms that exhibit selective RNAi silencing pan-neuronally^33^. Neuronal RNAi of *kin-1* also resulted in a decrease in the survival of worms under cold stress (Fig. 5d). Furthermore, we tested the effect of cAMP on survival in worms subjected to neuronal-specific *kin-1*(*RNAi*), indicating that cAMP can rescue the specific neuronal *kin-1*(*RNAi*) phenotype to cold stress (Fig. 5e). This result suggests that neural releasing cAMP is eventually transferred into the intestine.

**Figure 6:**
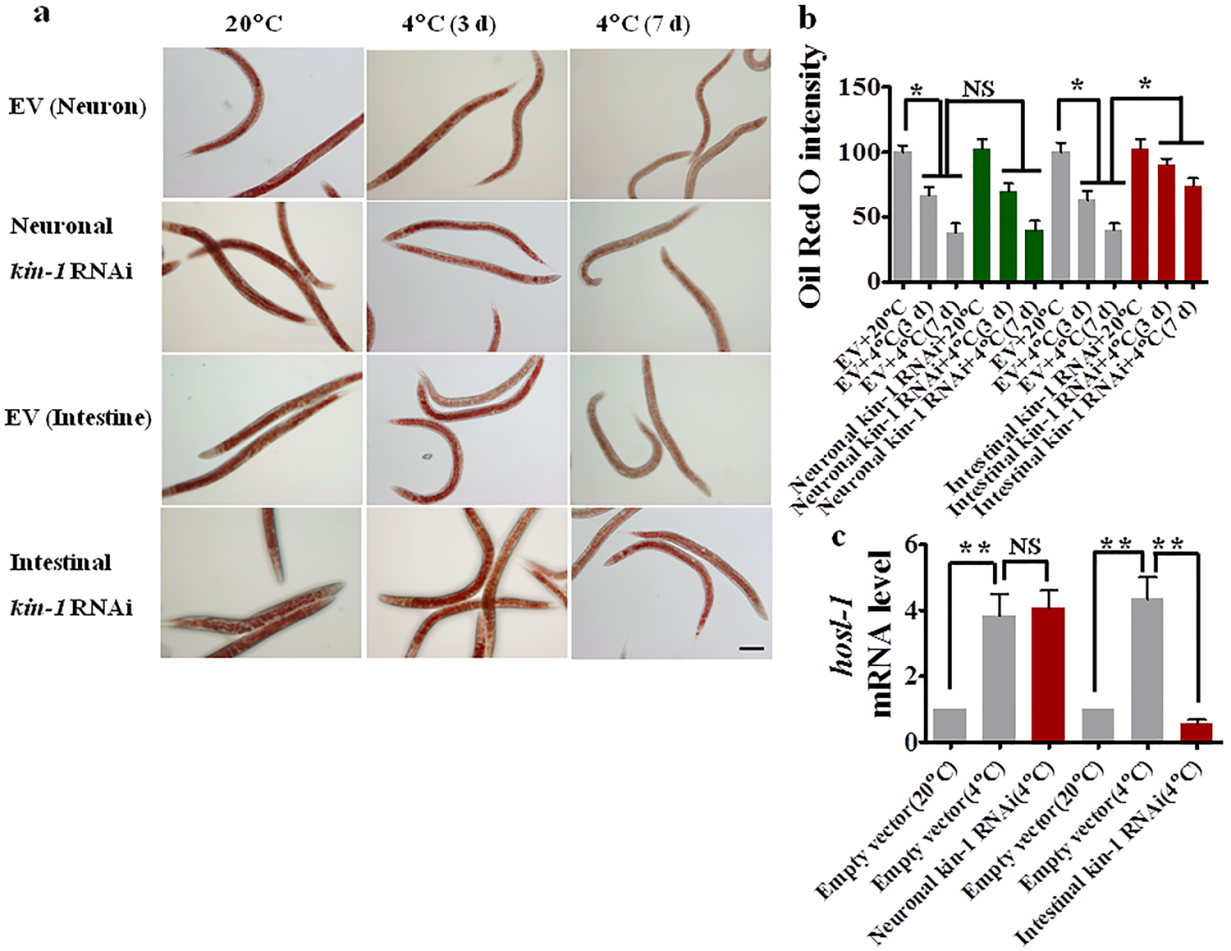
KIN-1/PKA in the intestine regulates the lipid hydrolysis during cold stress. (**a**) A representative image of lipid droplets using Oil Red O staining. Scale bar, 100 μm. (**b**) Lipid hydrolysis was significantly inhibited by intestinal-specific *kin-1*(*RNAi*). *P < 0.05. (**c**) The mRNA levels of *hosl-1* were down-regulated in worms subjected to intestinal-, but not neuronal-, specific *kin-1*(*RNAi*) after cold exposure. *P < 0.01.NS, not significant.

As *hosl-1* is a downstream effector of KIN-1, we tested the role of KIN-1 in specific tissues in the regulation of *hosl-1* expression. We found that intestinal-specific *kin-1*(*RNAi*) resulted in a significant decrease in either lipid hydrolysis or *hosl-1* mRNA levels under cold conditions (Fig. 6a–c). In contrast, neuronal-specific *kin-1*(*RNAi*) did not influence both lipid hydrolysis and *hosl-1* expression. Taken together, these results suggest that *kin-1* in the intestine is involved in cold tolerance by regulating *hosl-1* expression. *kin-1* in the neurons is also required for cold tolerance; however, the underlying mechanism remains unknown.

## Discussion

The Gsα-cAMP-PKA pathway that governs many facets of physiological functions is largely conserved in divergent organisms^34, 35^. For example, this signaling regulates lifespan and resistance to oxidative stress in yeast and mice^36^. The conserved nature of the PKA signaling pathway makes the worm an ideal model to investigate its role in cold tolerance. Our genetic and physiological analysis clearly indicates that the Gsα-cAMP-PKA signaling confers resistance to cold stress in worms. We show that KIN-1/PKA in the intestine performs its function in cold resistance by regulating the expression of *hosl-1* in the same tissue. Although KIN-1/PKA in the neurons is also required for cold tolerance; the underlying mechanism remains unknown. It should be noted that the components in the GSA-1 signaling pathway, such as *gsa-1* and *acy-1*, are mainly expressed in the neurons^29, 30^. It is conceivable that cAMP is produced and released in the neurons, and activates KIN-1/PKA in the neurons and intestine.

Another important finding is that cold stress induces fat mobilization via HOSL-1 lipase. *hosl-1*, a homolog of mammalian HSL, is identified by screening 20 genes in lipid metabolism pathways by RNAi. Genetic inactivation of *hosl-1* results in inhibition of lipid hydrolysis and increased susceptibility of worms to cold stress. The mechanism of HOSL-1 regulation in *C*. *elegans* remains largely unclear. In mammals, in response to cold stress, norepinephrine promotes lipolysis by activating HSL via the cAMP-PKA signaling in brown adipocytes^4^. *In vitro* experiment demonstrates that PKA either induces the expression of HSL or promotes its phosphorylation at Ser-660 and Ser-563, leading to an increase in its activity^37^. Our data indicate that PKA up-regulates the expression of *hosl-1*. As phosphorylation site prediction suggests that there are no conserved serine phosphorylation sites in HOSL-1, activation of HOSL-1 by phophorylation via PKA is unlikely in *C*. *elegans*. In mammals, the cAMP signalling pathway can activate the transcription factor CREB through PKA in a variety of cells^25, 38^, raising a possibility that PKA/KIN-1 up-regulates the expression of HSL/HOSL-1 through activating CREB/CRH-1. However, our data reveal that neither *crh-1*(*RNAi*) nor mutation in *crh-1*(*tz2*) influences the expression of *hosl-1* under cold conditions. Thereby, the mechanism underlying PKA/KIN-1 regulated the expression of *hosl-1* needs to be investigated further.

Although glycerol accumulation in response to cold stress is observed in invertebrates^11, 12^, very few studies have provided unambiguous evidence that glycerol directly contributes to cold tolerance. In this study, our results have indicated that *hosl-1*(*RNAi*), which results in a decrease in glycerol contents, is associated with shortened survival of cold stress, whereas exogenous application of glycerol extends survival of cold stress in both WT and *hosl-1* (RNAi) worms. In addition, a previous study has demonstrated that addition of glycerol substantially increases the glycerol levels in worm body^39^. These data suggest that glycerol accumulation due to lipid hydrolysis confers resistance to cold exposure in worms.

The cAMP-PKA-HSL signaling-mediated fat mobilization is required for resistance to cold stress in mammals^4^. Thus, the role of the cAMP-PKA-HSL signaling in cold tolerance is probably conserved from invertebrates to mammals. In mammals, fat mobilization in brown adipose tissues leads to the production of free fatty acids and glycerol under cold conditions. Finally, fatty acids is involved in the physiological activation of UCP1 for thermogenesis. As there are no UCP1 homologs in invertebrate genomes, invertebrates utilize glycerol to prevent cold insult under cold conditions. The molecular mechanism underlying fat mobilization-mediated cold tolerance in invertebrates needs to be further investigated.

## Materials and Methods

### Nematode strains

Standard conditions were used for *C*. *elegans* growth at 20 °C^40^. Mutations used in this study include: strains *acy-1*(*nu329*), *acy-2*(*ok3003*), *acy-3*(*tm2461*), *acy-4*(*tm2510*), CZ3086 (*kin-1*(*ok338*)/*mIs13I*), *kin-2*(*ce179*), *gsa-1*(*ce94*), *gsa-1*(*ce81*), *crh-1*(*tz2*), TU3401 (*sid-1*(*pk3321*)*V*; *uIs69V*) for neuronal-specific RNAi, NR222 (*rde-1*(*ne219*)*v*; *kzIs9*) for hypodermic-specific RNAi, NR350 (*rde-1*(*ne219*)*v*; *kzIs20*) for muscular-specific RNAi, BC14317(*dpy-5*(*e907*)*I*; *sEx14317*) (*Phosl-1*::*gfp*), and VP198 (*Pgpdh-1*::*gfp*) were kindly provided by the Caenorhabditis Genetics Center (CGC; http://www.cbs.umn.edu/CGC), which is funded by NIH Office of Research Infrastructure Programs (P40 OD010440). The strain *acy-1*(*tm5028*) was obtained from the National BioResource Project (NBRP). The strain MGH170 for intestinal-specific RNAi (*sid-1*(*qt9*); *Is* [*vha-6pr*::*sid-1*]; *Is* [*sur-5pr*::*GFPNLS*]) was kindly provided by Dr. Gary Ruvkun (Massachusetts General Hospital, Harvard Medical School). Mutants were backcrossed three times into the WT strain (N2) used in the laboratory. All strains were maintained on nematode growth media (NGM) and fed with *E*. *coli* strain OP50.

### Construction of transgenic strains

The vector expressing *Phosl-1*::*hosl-1*::*gfp* was constructed as follows: a 1552 bp of *hosl-1* promoter fragment was obtained by PCR on *C*. *elegans* genomic DNA using primers 5′-ACA TGC ATG CGA TTT GGA AAT GCG AGA CTC AGA C-3′ and 5′-ACG CGT CGA CAT TGT TTC AGT TTC AGA GAT T-3′ followed by SphI and SalI digestion. The fragment was inserted into pPD95.75 vector, resulting in the plasmid pP*hosl-1*. *hosl-1* cDNA was amplified by PCR using primers 5′-ACG CGT CGA CAT GCC GAA ACG AAA ATT CCG-3′ and 5′-CCC CCC GGG CTA TCG GGG CTC TTC ATT TA-3′ followed by SalI and XmaI digestion. The fragment was inserted into pPD95.75 vector, resulting in the plasmid expressing *Phosl-1*::*hosl-1*::*gfp*. This construct was co-injected with the marker plasmid pRF4 containing *rol-6*(*su1006*) into gonads of wild-type worms by standard techniques^41^.

### RNA Interference

The clones of genes for RNAi were from the Ahringer library^42^. The knockdown efficiency of RNAi was tested by using real-time PCR. As shown in Supplemental Tables 2 and 3, the expression of these genes was significantly reduced by RNAi. Furthermore, RNAi feeding experiments were performed on synchronized L1 larvae at 20 °C rather than at 4 °C.

### Cold tolerance assays

Synchronized populations of worms were cultivated at 20 °C until the young adult stage (i.e., within 12 h beyond the L4 stage). 50–60 young adult worms were transferred to 4 °C on the NGM plates and fed with *E*. *coli* strain OP50. As exposure to chill (4 °C) led to the defects in egg production, 5-fluoro-2′-deoxyuridine (FUDR) was not added into the assay plates. The number of living worms was counted at 24 h intervals. Immobile worms unresponsive to touch were scored as dead. Three plates of each genotype were performed per assay and all experiments were performed three times.

### cAMP measurement

cAMP concentration was measured as previously described^43^. After approximately 10,000 worms were collected and washed four times with M9 buffer, the worms were re-suspended in 0.1 M HCl to inactivate phosphodiesterase. After the worms were collected and centrifuged to form a loose pellet, the fluid surrounding the worms was removed. Worms were homogenized using Polytron-type homogenizer on ice, and the homogenates were sonicated. Then worm extracts were collected by centrifugation. The cAMP levels in the supernatant were determined using the cyclic AMP ELISA kit (Cayman Chemical, Ann Arbor, MI) according to the manufacturer’s instructions. Results were analyzed using 4 parametric logistic curve-fitting models.

### Fluorescence microscopy analysis of GFP-labeled worms

After 12 h of cold exposure (4 °C), the transgenic worms carrying *Phosl-1*::*hosl-1*::*gfp* and *Pgpdh-1*::*gfp* were immediately mounted in M9 onto microscope slides. The slides were viewed using a Zeiss Axioskop 2 Plus fluorescence microscope (Carl Zeiss, Jena, Germany) with a digital camera. Fluorescence intensity was quantified by using the Image J software (NIH). Three plates of about 30 animals per plate were tested per assay and all experiments were performed three times independently.

### Quantitative real-time PCR

Total RNA was extracted from worms with TRIzol Reagent (Invitrogen). Random-primed cDNAs were generated by reverse transcription of the total RNA samples with SuperScript II (Invitrogen). A real time-PCR analysis was conducted using SYBR Premix-Ex TagTM (Takara, Dalian, China) on an Applied Biosystems Prism 7000 Sequence Detection System (Applied Biosystems, Foster City, CA). *act-1* was used for an internal control. The primers used for PCR were as follows: *hosl-1*: 5′-GGC TCG CTC ATC AAC ACT GG-3′ (F), 5′-CAC CAT TTC TCC ACT CTT CC-3′ (R); *act-1*: 5′-CCA TCA TGA AGT GCG ACA TTG-3′ (F), 5′-CAT GGT TGA TGG GGC AAG AG-3′ (R).

### Oil Red O staining

Oil Red O staining was performed as previously described^44^. Briefly, after washed with PBS buffer, 200-300 worms were suspended in 120 μl of PBS. Then an equal volume of 2 × MRWB buffer (160 mM KCl, 40 mM NaCl, 14 mM Na_2_EGTA, 1 mM spermidine-HCl, 0.4 mM spermine, 30 mM Na-PIPES pH 7.4, 0.2% β-mercaptoethanol) containing 4% paraformaldehyde (PFA) was added. Samples were gently rocked for 1 h at room temperature. Animals were allowed to settle by gravity, buffer was aspirated. After washed with 1 × PBS to remove PFA, worms were resuspended in 60% isopropanol and incubated for 15 min at room temperature to dehydrate. Oil Red O was prepared as follows: a 0.5 g/100 ml isopropanol stock solution equilibrated for several days was freshly diluted to 60% with water and rocked for at least 1 h, then filtered with 0.22 µm-filter. Animals were allowed to settle, and isopropanol was removed. After 1 ml of 60% Oil-Red-O stain was added, animals were incubated overnight with rocking. Then, animals were mounted on a glass slide with an agarose pad (dissolve 2% agarose in 1 × PBS) and imaged with a Olympus color camera outfitted with DIC optics. Anterior intestinal cell areas were selected to determine Oil Red O intensity. Relative intensity was quantified using ImageJ software (NIH). All experiments were performed three times.

### Quantitative analysis of triacylglycerol (TAG)

TAG was measured using the method as described previously^45, 46^. Briefly, after approximately 10,000 worms were collected and washed four times with M9 buffer, the worms were incubated with 5 ml of ice-cold chloroform: methanol (1:1) overnight at −20 °C with occasional vortexing. Then 5 ml of a solution containing 0.2 M H_3_PO_4_ and 1 M KCl was added to the samples, resulting in separation of the organic and aqueous phases. The organic phase was collected and dried under argon, then resuspended in chloroform. The samples were loaded, and TLC plates were developed two thirds of the way up the plate in the first solvent system: chloroform: methanol: water: acetic acid (65:43:3:2.5), dried, and then the second solvent system: hexane: diethylether: acetic acid (80:20:2) was developed to the top of the plate. After spraying the plate with 0.005% primuline, lipids were visualized under UV light. The spots corresponding to TAG and the major phospholipids were scraped into labeled tubes. A known standard (15:0) was added into each tube as an internal standard, and then transesterified for GC/MS analysis to determine the fatty acid composition. The triglyceride (TAG) content was defined as the percentage of TAG in total lipids.

### Glycerol measurements

Glycerol content was measured as described previously with some modifications^47, 48^. Briefly, after worms were collected and centrifuged to form a loose pellet, the fluid surrounding the worms was removed. Then 0.5–1 ml of the pellet was dropped into liquid nitrogen by pipetting, and the frozen pellets were grounded with a mortar. After the powder was re-suspended in 400 μl RIPA solution, in soluble material was removed by centrifugation for 5 min at 10,000 rpm. The supernatant from each sample was divided, a portion of the supernatant was used for measurement of total protein content with a BCA protein assay kit (Pierce, Rockford, IL). And the rest was separated on a TLC plate. To locate the position of glycerol in samples, a glycerol standard was run on the same TLC plate. The TLC plate was developed to the top of the plate in the solvent system: chloroform: methanol (4:1). The spots corresponding to glycerol were scraped to determine glycerol content by the commercial kit (R-Biopharm). Glycerol levels were expressed relative to total protein content.

### Statistics

Differences in survival rates were analyzed using the log-rank test. Differences in gene expression were assessed by performing a one-way ANOVA followed by a Student-Newman-Keuls test. Data were analyzed using the SPSS11.0 software.

## Electronic supplementary material

Suppl Figures and Tables 2 and 3

Suppl Table 1
