# Supplementary material for: The cAMP-PKA pathway-mediated fat mobilization is required for cold tolerance in C. elegans
Source: Sci Rep. 2017 Apr 4;7:638. doi: 10.1038/s41598-017-00630-w (PMC5428847; doi:10.1038/s41598-017-00630-w)
Supplement: Supplementary file 1 — Suppl Figures and Tables 2 and 3 [file 41598_2017_630_MOESM1_ESM.pdf]

The cAMP-PKA pathway-mediated fat mobilization is required for cold tolerance in *C. elegans*

Fang Liu, Yi Xiao, Xing-Lai Ji, Ke-Qin Zhang, Cheng-Gang Zou

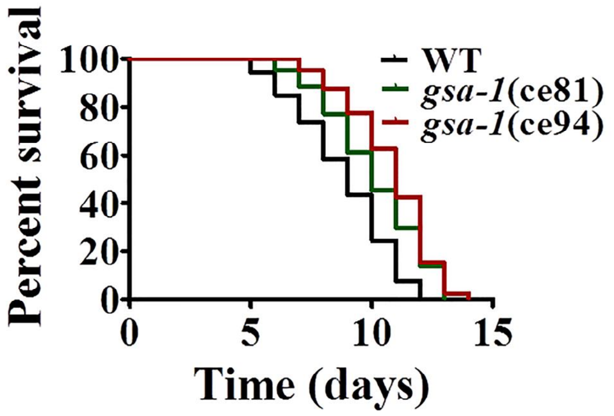

Supplementary Figure 1. Mutations in *gsa-1(ce94)* and *gsa-1(ce81)* promote survival of worms under cold conditions. $P < 0.01$ relative to wild type (WT) worms.

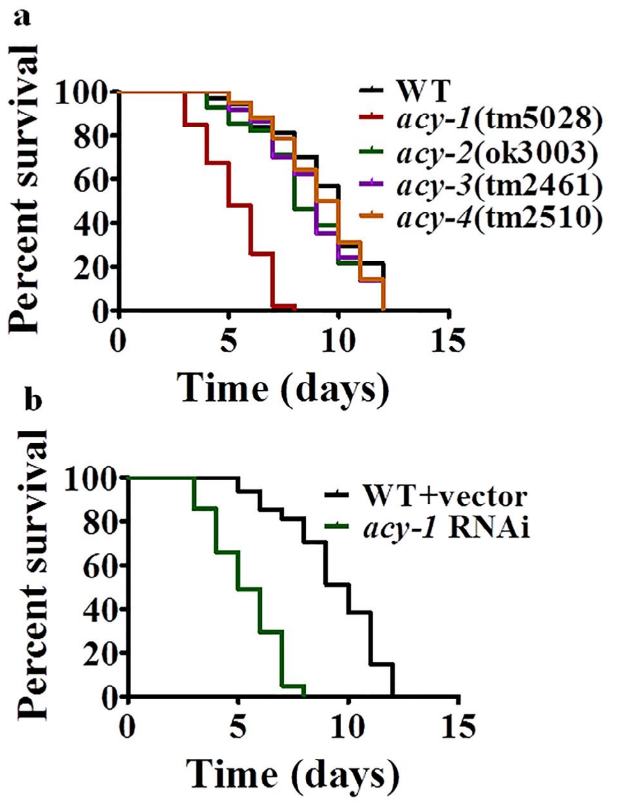

Supplementary Figure 2. Mutation or knockdown of *acy-1* reduces survival of worms under cold conditions. (a) $P < 0.001$ relative to wild type (WT) animals. (b) $P < 0.01$ relative to WT with empty vector.

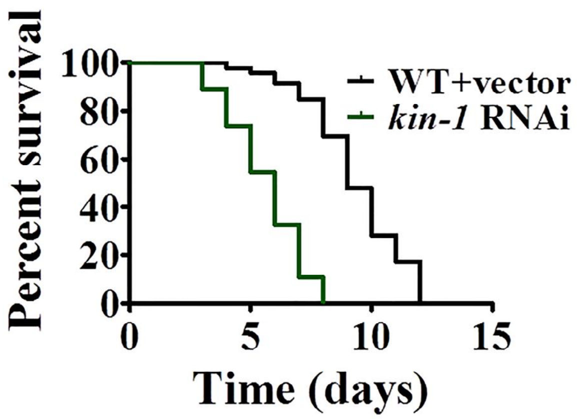

Supplementary Figure 3. Knockdown of *kin-1* by RNAi reduces survival of worms under cold conditions. $P < 0.01$ relative to wild type (WT) animals with empty vector.

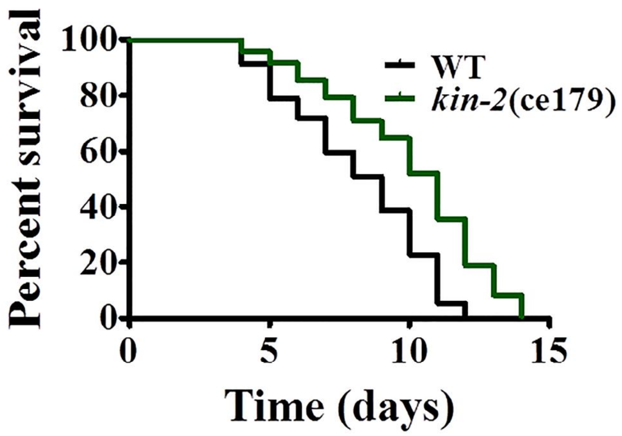

Supplementary Figure 4. Mutation in *kin-2(ce179)* promotes survival of worms under cold conditions. *P* < 0.01 relative to wild type (WT) worms.

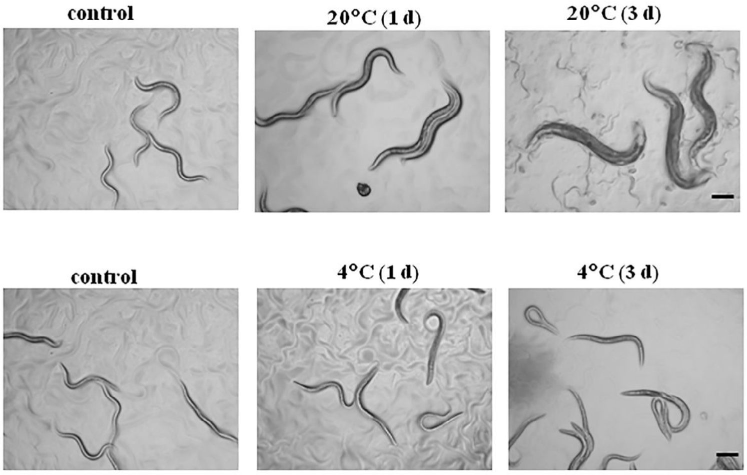

Supplementary Figure 5. Exposure to chill (4 °C) resulted the defects in egg production. Scale bar, 100 μm.

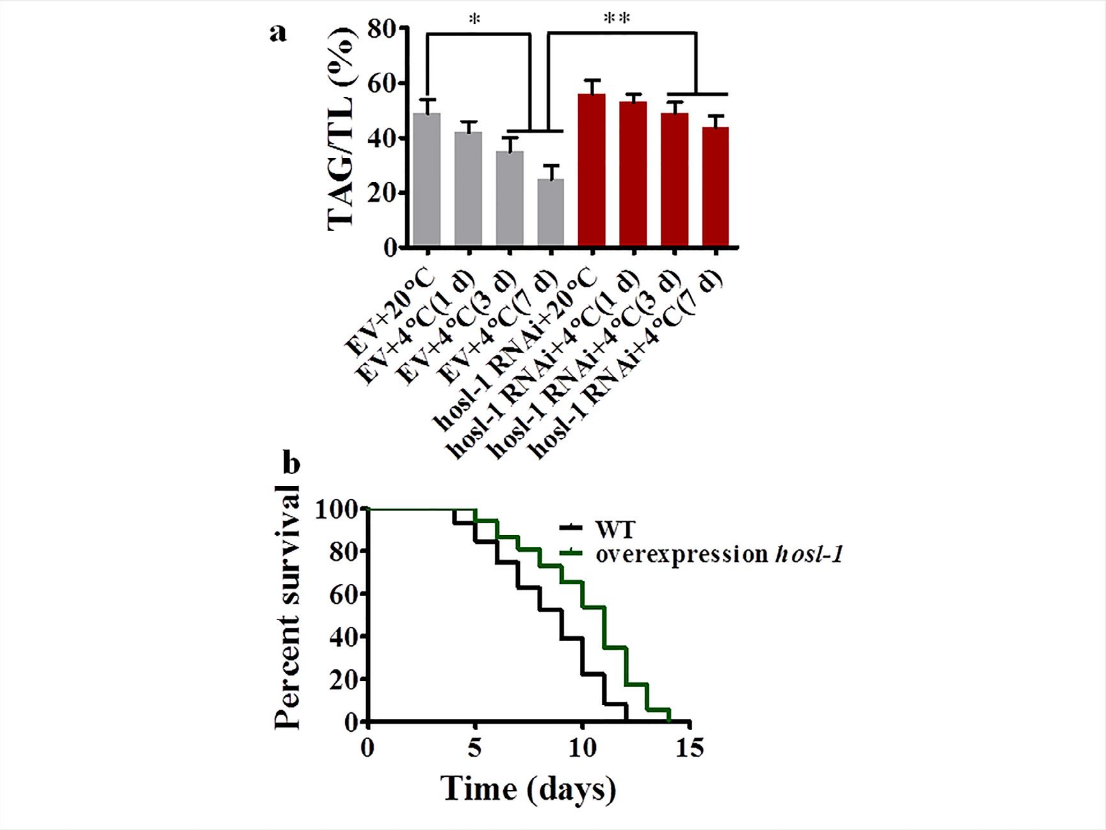

Supplementary Figure 6. Knockdown of *hosl-1* by RNAi inhibits cold-induced lipid hydrolysis. (a)The relative triacylglycerol contents were determined by GC-MS. **P*< 0.05; ***P*< 0.01. **Overexpression *hosl-1* in worms exhibited enhanced resistance to cold stress.** (b) *P* < 0.01 relative to wild type (WT) worms.

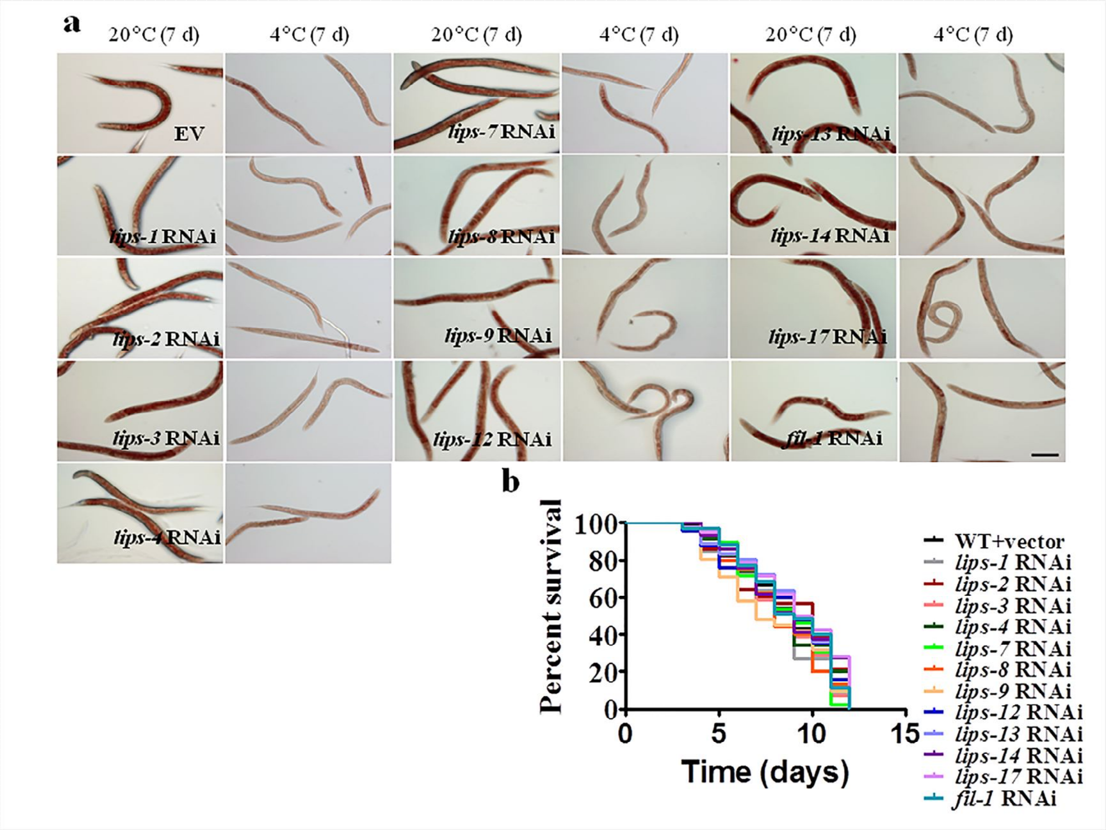

Supplementary Figure 7. Twelve class II lipase genes are not involved in lipid hydrolysis and cold tolerance. (a) Lipid content was determined by staining with Oil Red O. Scale bar, 150 µm. (b) Knockdown of these genes by RNAi did not influence survival of worms at 4°C.

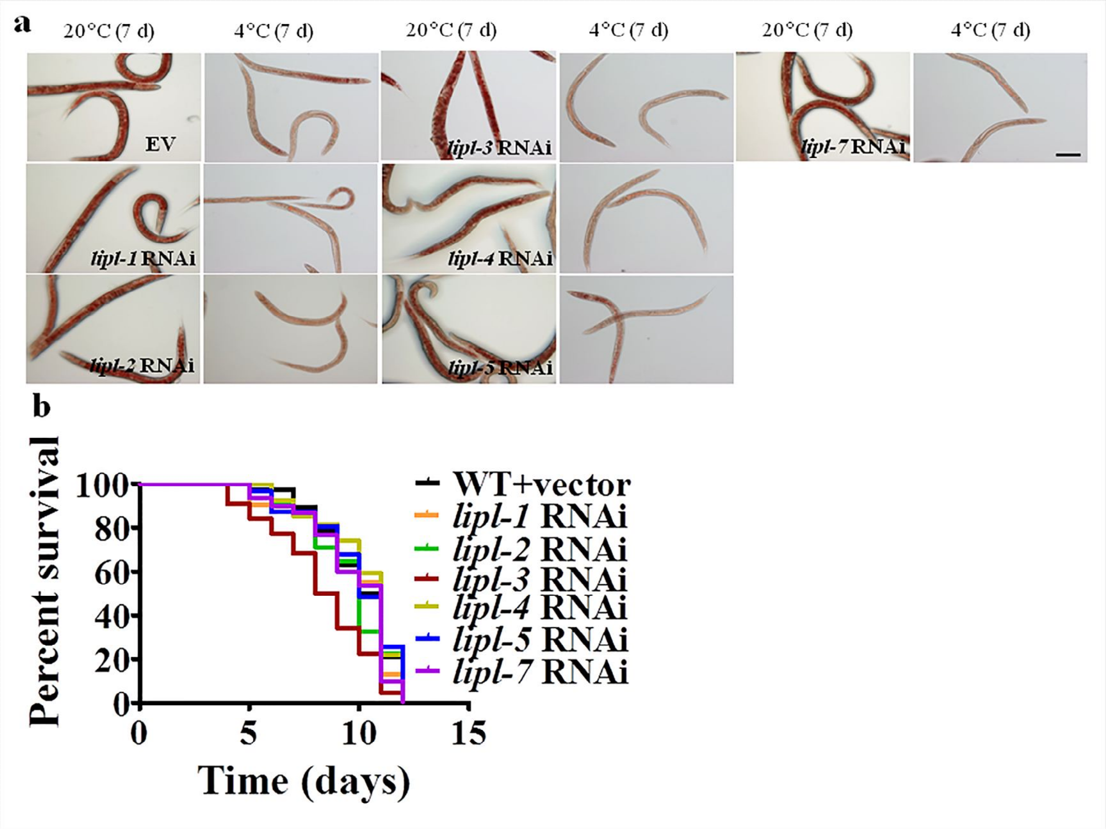

Supplementary Figure 8. Six lipase-related genes are not involved in lipid hydrolysis and cold tolerance. (a) Lipid content was determined by staining with Oil Red O. Scale bar, 150 μm. (b) Knockdown of these genes by RNAi did not influence survival of worms at 4°C.

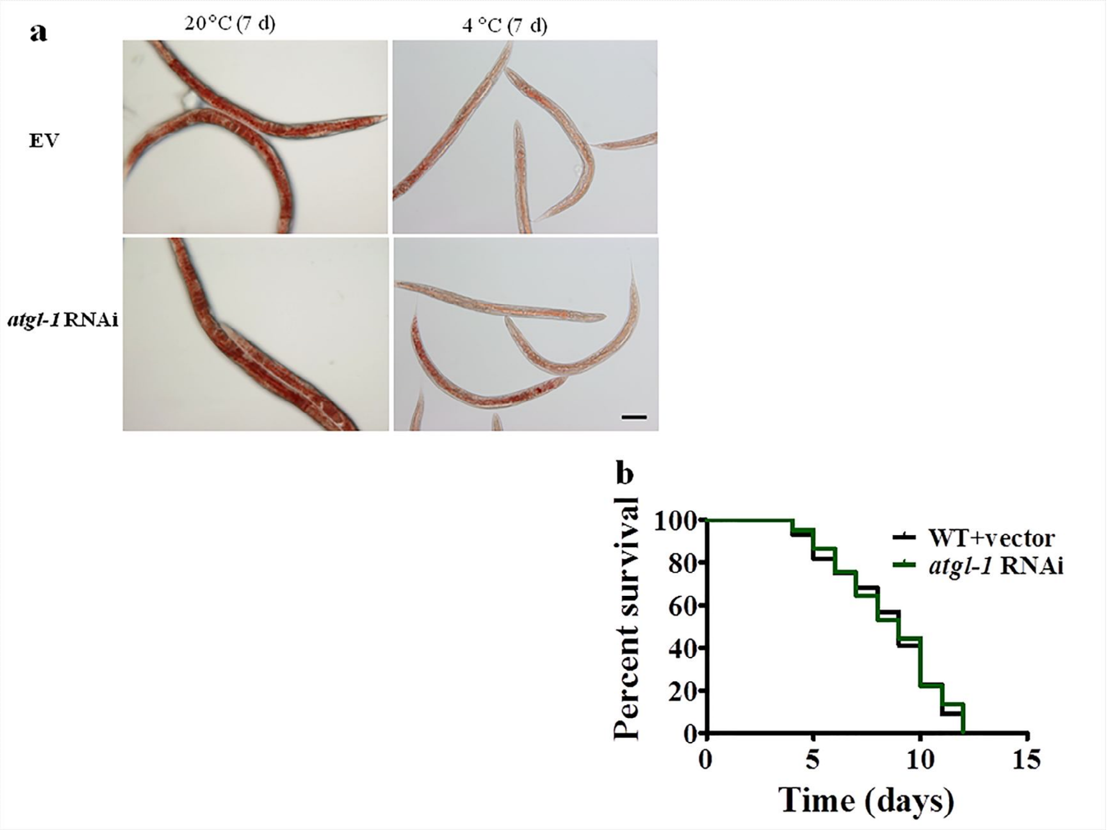

Supplementary Figure 9. *atgl-1* is not involved in lipid hydrolysis and cold tolerance. (a) Lipid content was determined by staining with Oil Red O. Scale bar, 100 µm. (b) Knockdown of *atgl-1* by RNAi did not influence survival of worms at 4°C.

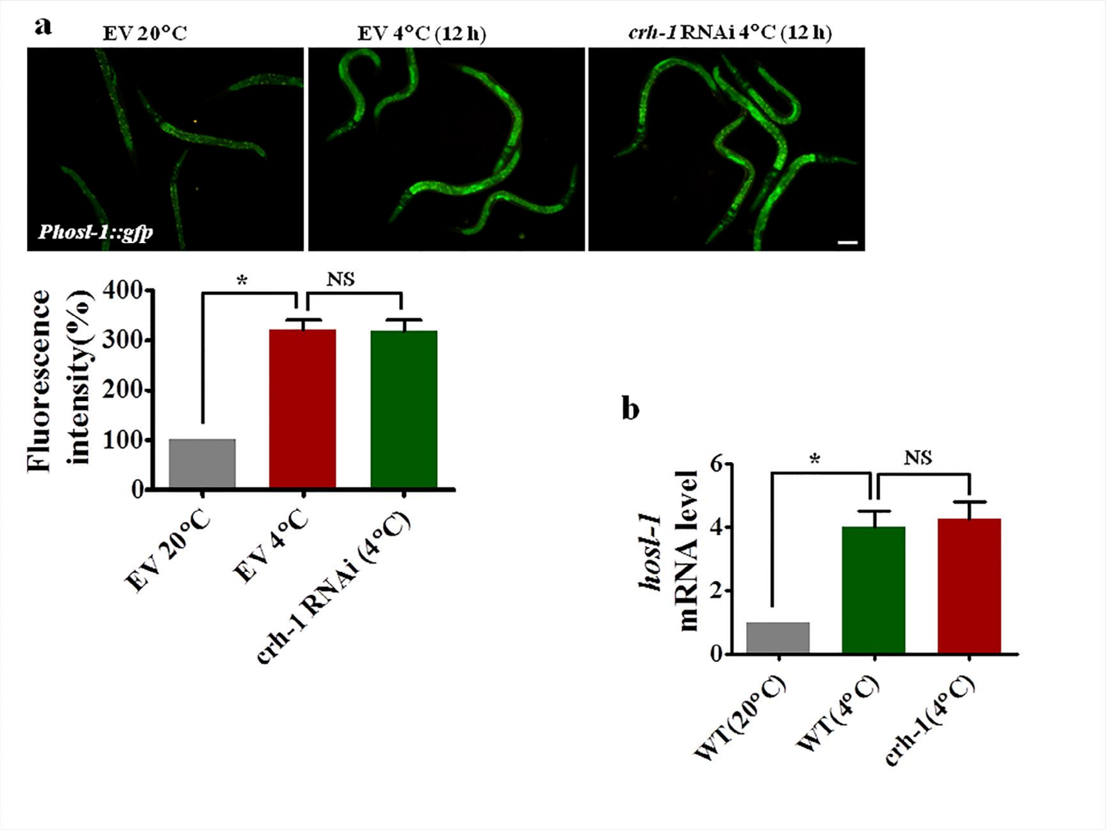

Supplementary Figure 10. CRH-1 is not involved in up-regulation of *hosl-1* expression mediated by KIN-1. (a) Knockdown of *crh-1* by RNAi did not influence the expression of *Phosl-1::gfp* at 4°C. Scale bar, 100 μm. (b) The mutation in *crh-1(tz2)* did not influence the mRNA levels of *hosl-1* at 4°C. **P*< 0.05; NS, not significant.

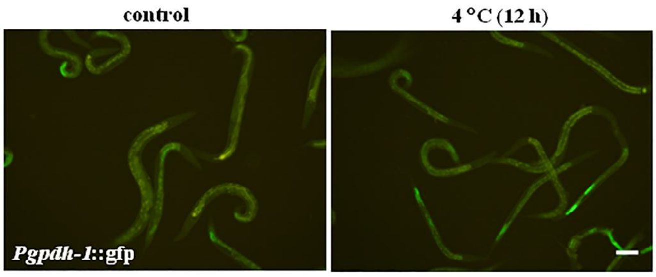

Supplementary Figure 11. Cold stress does not influence the expression of *gpdh-1*.

The expression of *Pgpdh-1::gfp* was not altered in worms exposed to cold (4 °C). Scale bar, 100 µm.

Supplementary Table 2. The efficiency of RNAi is detected by quantitative RT-PC.

Gene	The efficiency of RNAi
gsa-1	85%
acy-1	80%
kin-1	88%
hosl-1	81.5%
atgl-1	90%
lipl-1	75%
lipl-2	78%
lipl-3	90%
lipl-4	82%
lipl-5	78%
lipl-7	70%
lips-1	85%
lips-2	80%
lips-3	90%
lips-4	89%
lips-7	77%
lips-8	87%
lips-9	92%
lips-12	72%
lips-13	79%
lips-14	84%
lips-17	91%
fil-1	80%
crh-1	75%

Supplementary Table 3. Primer sequences used for quantitative RT-PC.

Gene	Sequences (5'→3')	Product length (bp)
gsa-1-F<br>*gsa-1*-R	TGCTGGAAAGACAAGGGAGT<br>ATCGTAGTTGTTCTGTCGCAC	117
acy-1-F<br>*acy-1*-R	GCCGCTCAATACCAAAGGTT<br>GCAACTGGAGTCATAAACGC	119
kin-1-F<br>*kin-1*-R	GAAGGACAACAAGAACTCGGC<br>GAATGATCCGGTTCCAAGG	145
hosl-1-F<br>*hosl-1*-R	GGCTCGCTCATCAACACTGG<br>CACCATTTCTCCACTCTTCC	231
atgl-1-F<br>*atgl-1*-R	CGGGTTGTGGATTCCTTTGTG<br>TGAGACCACAGGCAACGAT	126
lipl-1-F<br>*lipl-1*-R	TCCTGGAGCACGGTTATGTT<br>TTTGTGGCGTGGTCATCTTC	94
lipl-2-F<br>*lipl-2*-R	ACGGACAAGATACACCACCA<br>TCTCCAAGCCAATCATCGTCA	91
lipl-3-F<br>*lipl-3*-R	CTGTGACAATGTGTGCTTCCT<br>TCCGTGCCGAACCATCTGAA	142
lipl-4-F<br>*lipl-4*-R	GATGTGAATAATTCCCGTCC<br>GCCCAACCACACATCAAACC	135
lipl-5-F<br>*lipl-5*-R	GAGTGCTGGTTTCCTGTTCG<br>TCCCAACTCCAGTCCCAGAA	129
lipl-7-F<br>*lipl-7*-R	CAAATAGCCGTGGAACTCCTG<br>CCAAATCCACACTCGCTGTC	123
lips-1-F<br>*lips-1*-R	TCGGACCCAACCTATCGCAT<br>AGTCATTTCTGGGCAGGTGC	146
lips-2-F<br>*lips-2*-R	TCCCAGGCAGCAACATTTG<br>TTGGTCCGCTGCTGTTTCA	77
lips-3-F<br>*lips-3*-R	AAACTGTTCCCGCTTGGTGC<br>AGCGACCGTTCATTCCTGGA	258
lips-4-F<br>*lips-4*-R	GTGGCAAAGTTGATGTGTTCG<br>ATAGCCTCCACACGAATCTCC	137
lips-7-F<br>*lips-7*-R	CCTTCCTGTCAGTTGCTGGT<br>CCATCACGCTTCACAAATCCA	253
lips-8-F<br>*lips-8*-R	TTTGTTGGTGTTGCTGGGC<br>CAATCCGATAACCCTGCTGC	120
lips-9-F<br>*lips-9*-R	TCGGTTTCTCGCTTGGTGT<br>TGTCAGCGGTTCTCCAAGGT	92
lips-12-F<br>*lips-12*-R	GAAACCGTCTACGCAACATCA<br>CCCTTCTCCATACGCCACAAT	156
lips-13-F<br>*lips-13*-R	GGGCAATACAACCGACACTTC<br>GTGCCACAATATCAACCTGA	122
lips-14-F<br>*lips-14*-R	CATACAGCACAACTCACACCT<br>TGAACTTCGTCGGTAGGCAA	109

lips-17-F<br>*lips-17*-R	TGTATCTGTTGCTGGAGCC<br>CTTTATCGTCTCCTGGTCCGT	176
fil-1-F<br>*fil-1*-R	GGAGTTACCCTTGCCAGAAAG<br>AGACCCAAATCACAGGAACC	74
crh-1-F<br>*crh-1*-R	CGCCGTTGATGATGCTCTTG<br>TCACCTTATCCGCCGTTTCT	141
